# Mechanisms of lung endothelial cell injury and survival in pulmonary arterial hypertension

**DOI:** 10.1152/ajplung.00208.2024

**Published:** 2024-10-15

**Authors:** Ygor Marinho, Elizabeth S. Villarreal, Omar Loya, Suellen D. Oliveira

**Affiliations:** ^1^Vascular Immunobiology Lab, Department of Anesthesiology, College of Medicine, University of Illinois Chicago, Chicago, Illinois, United States; ^2^Department of Physiology and Biophysics, College of Medicine, University of Illinois Chicago, Chicago, Illinois, United States

**Keywords:** apoptosis, endothelial cell, endothelial dysfunction, endothelial-to-mesenchymal transition, pulmonary arterial hypertension

## Abstract

Pulmonary arterial hypertension (PAH) is a progressive, chronic, and incurable inflammatory pulmonary vascular disease characterized by significant sex bias and largely unexplored microbial-associated molecular mechanisms that may influence its development and sex prevalence across various subgroups. PAH can be subclassified as idiopathic, heritable, or associated with conditions such as connective tissue diseases, congenital heart defects, liver disease, infections, and chronic exposure to drugs or toxins. During PAH progression, lung vascular endothelial cells (ECs) undergo dramatic morphofunctional transformations in response to acute and chronic inflammation. These transformations include the appearance and expansion of abnormal vascular cell phenotypes such as those derived from apoptosis-resistant cell growth and endothelial-to-mesenchymal transition (EndoMT). Compelling evidence indicates that these endothelial phenotypes seem to be triggered by chronic lung vascular injury and dysfunction, often characterized by reduced secretion of vasoactive molecules like nitric oxide (NO) and exacerbated response to vasoconstrictors such as Endothelin-1 (ET-1), both long-term known contributors of PAH pathogenesis. This review sheds light on the mechanisms of EC dysfunction, apoptosis, and EndoMT in PAH, aiming to unravel the intricate interactions between ECs, pathogens, and other cell types that drive the onset and progression of this devastating disease. Ultimately, we hope to provide an overview of the complex functions of lung vascular ECs in PAH, inspiring novel therapeutic strategies that target these dysfunctional cells to improve the treatment landscape for PAH, particularly in the face of current and emerging global pathogenic threats.

## Graphical abstract

**Figure:**
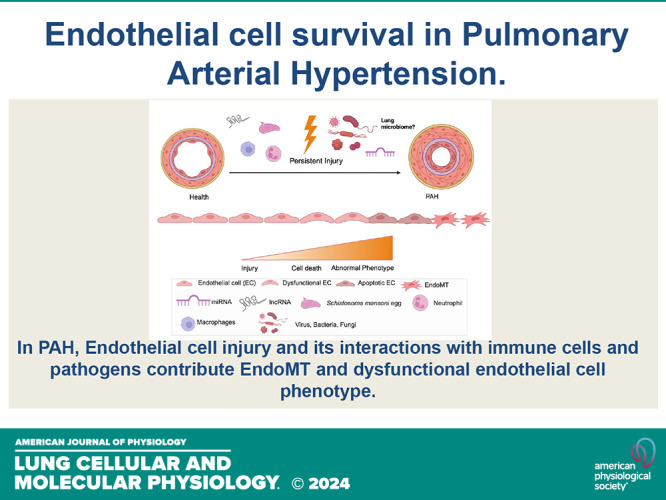

## INTRODUCTION

Pulmonary arterial hypertension (PAH), classified as group 1 pulmonary hypertension (PH), is a severe cardiopulmonary disease with no cure characterized by increased mean pulmonary arterial pressure (mPAP), pulmonary artery wedge pressure (PAWP) ≤ 15 mm/Hg, and right ventricular hypertrophy (RVH), which can lead to right ventricle (RV) failure and premature death (1, 2). Due to its multifactorial aspect, PAH may emerge from multiple conditions, including an unknown (idiopathic) or genetic (heritable) cause or as a consequence of liver, congenital heart, or connective disease (1). Moreover, PAH can also evolve as a chronic complication of drug/toxin exposure and infection with the human immunodeficiency virus (HIV) and/or with the intravascular parasite of the genus *Schistosoma*, known to cause schistosomiasis (1, 3). Data from the PH Association Registry (PHAR) indicate that the mortality rate for PAH ranges from 8% to 21% within the first three years of diagnosis (4). Furthermore, despite being more prevalent in women, male patients with PAH tend to have a worse disease prognosis. This disparity is partly attributed to the effects of sex hormones (5, 6) and underscores the urgent need for a better understanding of how sex differences affect disease onset and progression, and the development of sex-specific therapies for a more personalized future treatment of this life-threatening illness.

During PAH, elevated mPAP occurs in response to increased vasoconstriction, progressive remodeling, and eventual obstruction of the pulmonary arteries, mainly rising from chronic inflammation, migration, and hyperproliferation of cells, including endothelial cells (ECs) and smooth muscle cells (SMCs) within the lung vasculature (2, 7, 8). Indeed, chronic lung EC injury and selective apoptosis seem to contribute to the expansion of an abnormal cell phenotype, which can result from apoptosis-resistance cell growth and endothelial-to-mesenchymal transition (EndoMT)—a process by which EC acquires mesenchymal or “muscle-like” characteristics (9, 10). This EC-PAH associated dysfunctional profile is primarily characterized by defective synthesis and secretion of the vasoactive molecules, such as the vasodilator nitric oxide (NO), triggered by several factors, including hypoxia, shear stress, and persistent exposure to inflammatory mediators (11, 12). Besides impacting the normal vascular physiology by reducing the levels of NO, many of these factors can also lead to massive secretion of potent vasoconstrictors, including endothelin-1 (ET-1), which greatly increases the vascular resistance within the pulmonary circulation (12).

Beyond ECs, other vascular cells like SMCs, fibroblasts, and pericytes, as well as nonvascular cells, such as macrophages, lymphocytes, and neutrophils, are also known to contribute to PAH progression. Moreover, the direct or indirect crosstalk among these cell types is crucial for maintaining lung vascular homeostasis and sustaining healthy endothelial survival against damage-associated molecular patterns (DAMPs) and pathogen-associated molecular patterns (PAMPs), although it can also exacerbate the inflammatory response that contributes to the progression of the disease. Generally, ECs primarily recognizes PAMPs and DAMPs via activation of pattern recognition receptors (PRRs) such as Toll-like receptors (TLRs), which aid their survival in response to infection by bacteria (TLR2 and TLR4), viruses (TLR3, TLR7 and TLR8), and even fungi (TLR2) and parasites, including *Schistosoma spp* (TLR4) (13, 14). However, knowledge about how parasites and microorganisms in general contribute to vascular pulmonary injury and selective EC death in PAH remains in need of significant research efforts. Therefore, this comprehensive review explores the recently reported biological mechanisms underlying EC survival in pulmonary vascular disease, specifically PAH, shedding light on processes such as cell hyperproliferation and dysfunction. Moreover, we highlight how lung EC interaction with pathogens, the microbiota, and other cell types contributes to PAH onset and progression.

## METHODS

This comprehensive review was conducted by searching the PubMed database for free full research publications within the last five years (2019–2024), excluding review articles (with the exception of key references needed for full topic comprehension). Literature research was conducted by using keywords, such as endothelial cell, apoptosis, endothelial dysfunction, endothelial-to-mesenchymal transition, and pulmonary arterial hypertension. The literature research was completed by July 2024.

## LUNG ECs: FROM INJURY TO ABNORMAL PHENOTYPES IN PAH

### EC Apoptosis Mechanism in PAH: an Overview

In the initial stages of PAH, several reports indicated that lung vascular ECs are more susceptible to apoptosis, but as the disease progresses, a hyperproliferative and apoptosis-resistant phenotype emerges, contributing to progressive vascular remodeling and, often, microvascular obstruction (10). Many intrinsic and extrinsic factors are responsible for modulating lung EC homeostasis and phenotypes, including transcription factors, sex, inflammatory mediators, mutation, and depletion of receptors such as the bone morphogenetic protein receptor type II (BMPR2), microRNAs (miRNAs), and long noncoding RNAs (lncRNAs). In terms of transcription factors, elevated tumor suppressor p53 has been implicated in lung EC apoptosis in the monocrotaline (MCT)-induced PH model (15), by regulating Bax (pro-apoptotic protein) and Bcl-2 (antiapoptotic protein). Specifically, it was observed that increased p53 contributes to elevated Bax/Bcl-2 ratio (15), indicative of apoptosis, which may exacerbate the disease over time. Whole transcriptome sequencing by Florentin et al. (8) also identified amphiregulin (*AREG*) as an EC survival factor. Moreover, it was observed that pulmonary artery ECs transfected with siRNA against *AREG* showed increased apoptosis under hypoxia, measured by an increase in caspase 3 (8). Specifically, the downregulation of *AREG*, along with its receptor, the epidermal growth factor receptor (EGFR), exacerbated PH pathogenesis in animal models of hypoxia-induced PH (8).

Recent observations also indicate that EC death can be impacted by biological sex. Specifically, the Y-Chromosome gene [ubiquitously transcribed tetratricopeptide repeat containing, Y-linked (Uty)] knockdown, performed by intratracheal instillation of siRNA (Si-uty) in gonadectomized male mice exposed to hypoxia, promoted an increase in RV systolic pressure (RVSP) compared with controls. In addition, RNA sequencing on lung tissue from hypoxia control compared with the Si-Uty mouse model identified elevated expression of the pro-apoptotic chemokines CXCL9 and CXCL10, known to induce EC apoptosis. Interestingly, both chemokines were significantly upregulated, mainly in the lungs of the female patients with PAH (6). Opposing these observations, treatment with sex hormones, particularly estradiol, demonstrated a protective effect against lung EC apoptosis by significantly blocking caspase3 cleavage and activity within the lungs of Sprague-Dawley hyperresponsive rats (SDHR) in response to SU5416-induced vascular injury—an effect lost after four weeks post-SU5416 (16). Sex differences can also play a role in necrotic lung EC death characterized by cell swelling, membrane rupture, high mobility group box 1 (HMGB1) expression, and inflammation due to acute injury or stress (17, 18). Particularly, Rafikov et al. (17) observed a significant increase in pulmonary vascular necrosis in the lungs of male PH rats and PAH patients compared with females. Altogether, these data reinforce the importance of better understanding the contribution of biological sex and hormonal therapies for healthy vascular cell survival, including survival of lung ECs.

As a disease with a strong inflammatory component, the interaction of immune cell-derived factors with lung ECs also plays an essential role in PAH development, including significantly influencing EC fate. For instance, while proinflammatory factors such as neutrophilic elastase (NE) (19) and classical M1 macrophage-derived cytokines (20) substantially contribute to disease onset by triggering EC apoptosis, anti-inflammatory M2 macrophages have been largely linked to the hyperproliferative phase of the disease (20). In line with these findings, peptidyl-prolyl isomerase (Pin1: an important driver of proliferation and inflammation) was found to be increased in the lung tissue of patients with PAH, cultured human pulmonary microvascular ECs, and animal models, with its inhibition being able to reverse pulmonary vascular remodeling by interacting with members of the transforming growth factor β (TGF-β)/bone morphogenetic protein (BMP)-mediated signaling (21). Indeed, depletion of canonical BMP members such as BMPR2 has been largely implicated in several subgroups of PAH, including in Schistosomiasis-associated PAH (Sch-PH) (14). Beyond pathogens, many molecular mechanisms have been described to be able to decrease BMPR2 expression, including overexpression of endothelium-derived autocrine molecules as the angiocrine factor inhibin-β-A (22). Despite the robust role of BMPR2 in PAH, specific pharmacological or biotherapeutic alternatives to fully restore its lung EC-mediated canonical signaling remain challenging, especially when translating findings from rodent animal models into the complexities of human patients’ physiology. As a recent example, knocking down BMPR2 expression in human lung microvascular ECs impaired the function of the water channel aquaporin-1 (AQP1) (23), whereas its upregulation also induced EC proliferation in the SU5416/Hypoxia PH rat model (24). The contribution of AQP1 in EC apoptosis is complex and literature indicates it may be experimentally model-related; however, it was described that inhibition of AQP1 by Bacopaside II, derived from medicinal plant *Bacopa monnieri,* contributed to concentration-dependent apoptosis (25).

Besides factors and receptors, EC apoptosis can also be regulated by eicosanoids and conserved small noncoding (miRNAs) and long noncoding RNAs (lncRNAs). In terms of recent findings on eicosanoids, Ruffenach et al. (26) observed that 15-hydroxyeicosatetraenoic acid (15-HETE), a vasoconstrictor and proliferative promoter, led to significant apoptosis in lung ECs of patients with PAH and animal models of PH (27). Similarly, increased circulating miR-let-7i-5p and reduced miRNA-320a, pivotal for cell proliferation and migration, were observed in the plasma of patients with idiopathic PAH (IPAH), with network construction for target genes revealing their role in the transcriptional regulation of 11–20 genes implicated in lung EC function (28). In addition, miR-30a-5p overexpression was described to promote growth and prevent apoptosis of human pulmonary artery endothelial cells (PAECs) under hypoxia (29). In another study, Yang et al. (30) also observed a significant increase in plasma lncRNA-TCONS_00008552 of patients with PAH, although its functional role was not further explored. These data reinforce the fact that miRNAs and lncRNAs may be leveraged as putative disease biomarkers and as molecular targets for future therapeutic approaches to prevent lung EC death or revert its homeostasis in PAH, although further research remains required.

In summary, EC apoptosis contributes to PAH development by multiple factors such as those described earlier, specifically by facilitating the expansion of a pool of hyperproliferative and apoptosis-resistant cell phenotypes that leads to progressive pulmonary vascular remodeling and obstruction. Furthermore, understanding the molecular mechanism that triggers and maintains lung EC apoptosis in PAH may open novel therapeutic avenues. Specifically, antiapoptotic EC-targeted therapies have the potential to rescue pulmonary vascular homeostasis by reverting the sustained vascular injury that contributes to PAH’s onset and progression.

### Endothelial-to-Mesenchymal Transition

EndoMT, a process by which ECs acquire mesenchymal characteristics (9), contributes to the abnormal lung EC phenotype observed in PAH. This transition is substantially induced by TGF-β and nuclear translocation of β-catenin resulting in decreased expression of endothelial markers, such as VE-Cadherin and CD31, simultaneous with a concomitant gain of mesenchymal or fibroblast-like characteristics such as α-smooth muscle actin (α-SMA) and N-cadherin expression (9, 31, 32). In line with these observations, transcription factors such as Twist have played a major role in EndoMT. Increased Twist1 expression was found in the lungs of patients with PAH, mediating EndoMT by increasing TGF-β expression in ECs, and its deletion using a Tie2-specific Twist1 conditional knockout mice attenuated the accumulation of α-SMA-positive cells in the pulmonary arterioles (33). In addition, increased expression of the transcription factor hypoxia-inducible factor-2α (HIF-2α) has also been implicated in EndoMT observed in patients with IPAH and animals under experimental PH (34). In contrast, overexpression of the family with sequence similarity 13, member A (*FAM13A*) gene, has been shown to inhibit the induction of mesenchymal markers during EndoMT in PAH-associated ECs, likely by repressing β-catenin-mediated signaling (35). In addition to *FAM13A* overexpression, in vitro inhibition of integrin-ανβ5 significantly reduced *ACTA2* gene expression, known to encode the mesenchymal marker α-SMA, in human lung microvascular ECs mitigating in part TGF-β-induced EndoMT gene expression. However, blocking of integrin‐ανβ5 in vivo in chronic hypoxia rat model of PH exacerbated the disease severity (9).

TGF-β-mediated EndoMT can be antagonized by BMP-mediated signaling (36). In patients with PAH, BMPR2 and BMPR Type 1 A are significantly reduced in the lungs (32, 37), and its specific knockdown in lung ECs also unveiled a mesenchymal identity (32, 36). Restoring lung BMPR2-mediated signaling using BMP receptor ligand 9 (BMP9) prevented EC death and reversed PAH in animals carrying the human BMPR2 loss-of-function mutation, *R899X* (38). However, the long-term treatment with the same ligand was described to also reduce VE-cadherin expression in patient-derived microvascular ECs, leading to a classical EndoMT-like elongated morphology (39). These observations reinforce the need to further understand how BMP-related signaling pathways affecting lung EC survival converge into EndoMT and which anti-inflammatory sensors are able to prevent or reverse it. In terms of anti-inflammatory stress sensors, it was observed that peroxisome proliferator-activated receptor gamma coactivator 1α (PGC-1α) overexpression in PAECs significantly blocked EndoMT, indicating that PGC-1α inhibition may protect against PAH development by preserving lung EC function and reducing pulmonary vascular remodeling (31). Other anti-inflammatory molecules, such as Paeoniflorin (a monoterpene glucoside) and Naringin (a compound found in citrus fruits), may also offer a therapeutic potential. Recent studies published in PAH indicate that Naringin and Paeoniflorin offer protection against acute lung injury and EC injury induced by lipopolysaccharide, hydrogen peroxide, or radiation, thus ameliorating EndoMT (40–42).

**Figure 1.**
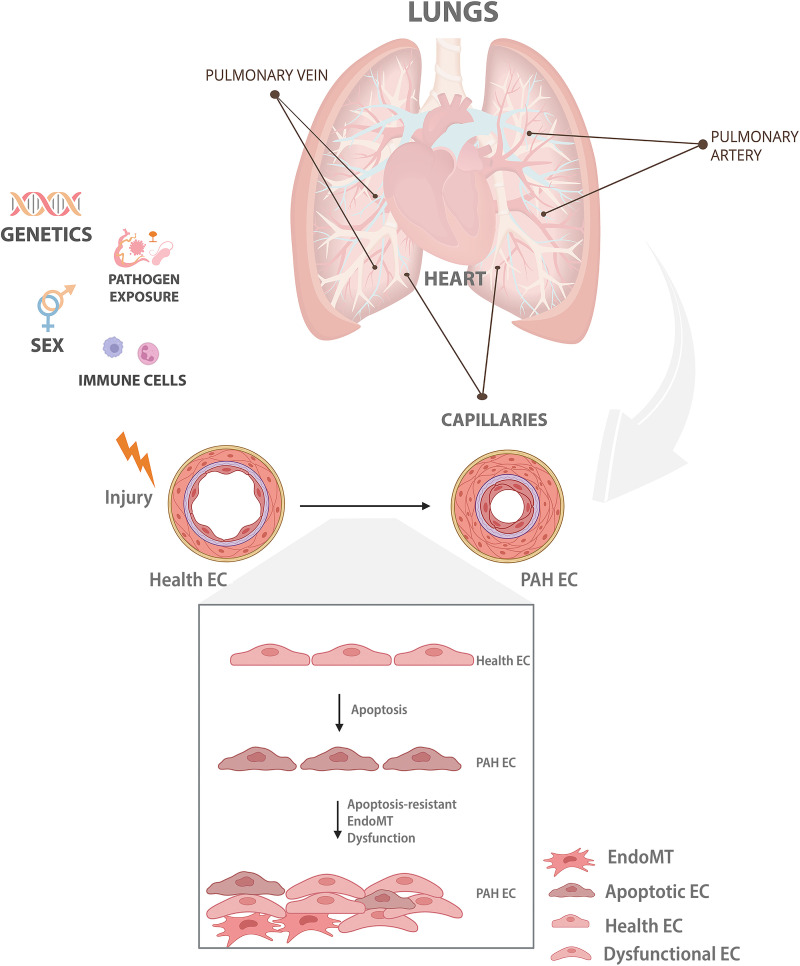
Endothelial cell (EC) injury and vascular remodeling in pulmonary arterial hypertension (PAH). Multiple genetic and epigenetic, including pathogens, sex, inflammation, and direct crosstalk with immune cells can induce/regulate injury of healthy ECs, leading to cell death and the expansion of an abnormal EC phenotype likely able to sustain vascular inflammation, resulting in prolonged vasoconstriction and the development of severe pulmonary vascular remodeling over time. In PAH, these phenotypes have been associated with EC dysfunction, apoptosis resistance, and endothelial-to-mesenchymal transition (EndoMT). Figure created with BioRender.com.

In summary, lung EC death/survival and EndoMT can be induced/regulated by multiple genetic and epigenetic factors, including inflammation, pathogens, sex, transcription factors, miRNAs, and lncRNAs, and also through its direct crosstalk with immune cells, which over time completely transforms the lung vasculature from a low resistance and high-perfusion network into an inflamed, remodeled, and stiff environment (Fig. 1).

## LUNG EC DYSFUNCTION: DOES IT PRECEDE EC SELECTIVE SURVIVAL?

### EC Dysfunction: Endothelial Nitric Oxide Synthase-Derived Nitric Oxide and Reactive Nitrogen Species

EC regulates vascular function by synthesizing and secreting various molecules, including vasoactive NO and ET-1 (12). Defective EC-derived NO production stands as a primary factor of endothelial dysfunction in cardiovascular and pulmonary diseases affecting the vasculature. In the quiescent endothelium, NO mainly derives from the constitutive enzyme endothelial nitric oxide synthase (eNOS), which utilizes l-arginine as its substrate. Once released, NO diffuses into vascular SMCs acting as a vasodilator (43); however, during inflammation, dysfunctional ECs can undergo a process called “*eNOS uncoupling*” in which dimeric eNOS becomes monomeric (i.e., uncoupled), leading to the production of the highly reactive superoxide anion $O_{2}^{-}$. $O_{2}^{-}$ can react with NO-yielding peroxynitrite (ONOO^−^) (11), which has a vasoconstrictive effect (44). Therefore, uncoupled eNOS contributes to impairment in the endothelium-dependent vascular relaxation, which is a clear sign of EC dysfunction observed in PAH progression (45).

Reduced EC-derived NO can also lead to increased tissue inflammation (46), further stimulating the search for novel curative treatments to prevent eNOS uncoupling and/or increase NO bioavailability. Recent findings explored a possible treatment to recouple eNOS using Amorphous nano-selenium quantum dots (A-SeQDs). Interestingly, in the MCT-induced PH model, animals that received A-SeQD treatment exhibited significantly lower RVSP and RVH, demonstrating that recoupling eNOS remains an important avenue for future therapeutic approaches in PAH (47).

### EC Dysfunction: a Brief Discussion on the Role of ET-1 Expression and Inhibition

Besides eNOS uncoupling, elevated ET-1, a small protein with strong vasoconstricting properties, has been classically linked to EC dysfunction. ET-1 promotes signaling via two receptors: ET_A_ and ET_B_. Whereas activation of ET_A_ and ET_B_ on SMCs mediates vasoconstriction, ET-1 binding of ET_B_ on EC produces vasodilation via NO secretion (48). Activated ET_B_ receptors in EC and immune macrophages are also known to promote the clearing of extracellular ET-1 levels, which aids in the decrease of blood pressure and helps maintain normal vascular function (48, 49). Exhibiting both a potent vasoconstrictor effect and EndoMT-associated potential (50), elevated ET-1 levels have been strongly linked to the pathogenesis of PAH. Indeed, ET-1-targeted therapies, as with competitive inhibitor Bosentan, not only reduce vasoconstriction but slow the proliferation of PASMCs and the overall progression of PAH in some patients (51). In recent studies using hypoxia-induced PH animal models, Bosentan treatment increased EC function through increased eNOS protein expression as well as inhibited bone marrow-derived macrophage function in the lungs. The increased abundance of EC through Bosentan therapy reveals an effect contrasting the pro-inflammatory and vasoconstriction observed in PAH. For example, inhibition of ET_A_ in lung ECs by Bosentan could be associated with the upregulation of eNOS, which leads to increased NO and dilation of the blood vessels. In addition, the suppression of bone marrow-derived macrophage function occurred through the inhibition of ET_A_ by downregulating the expression of IL-6, a multifunctional cytokine that has pro-inflammatory effects in PAH, as well as by downregulating the expression level of the monocyte chemoattractant protein-1 (MCP1). Altogether, the Bosentan effect in simultaneously influencing lung EC function and bone marrow-derived cell kinetics indicates Bosentan’s multiple mechanisms of action to ameliorate PAH (52).

**Figure 2.**
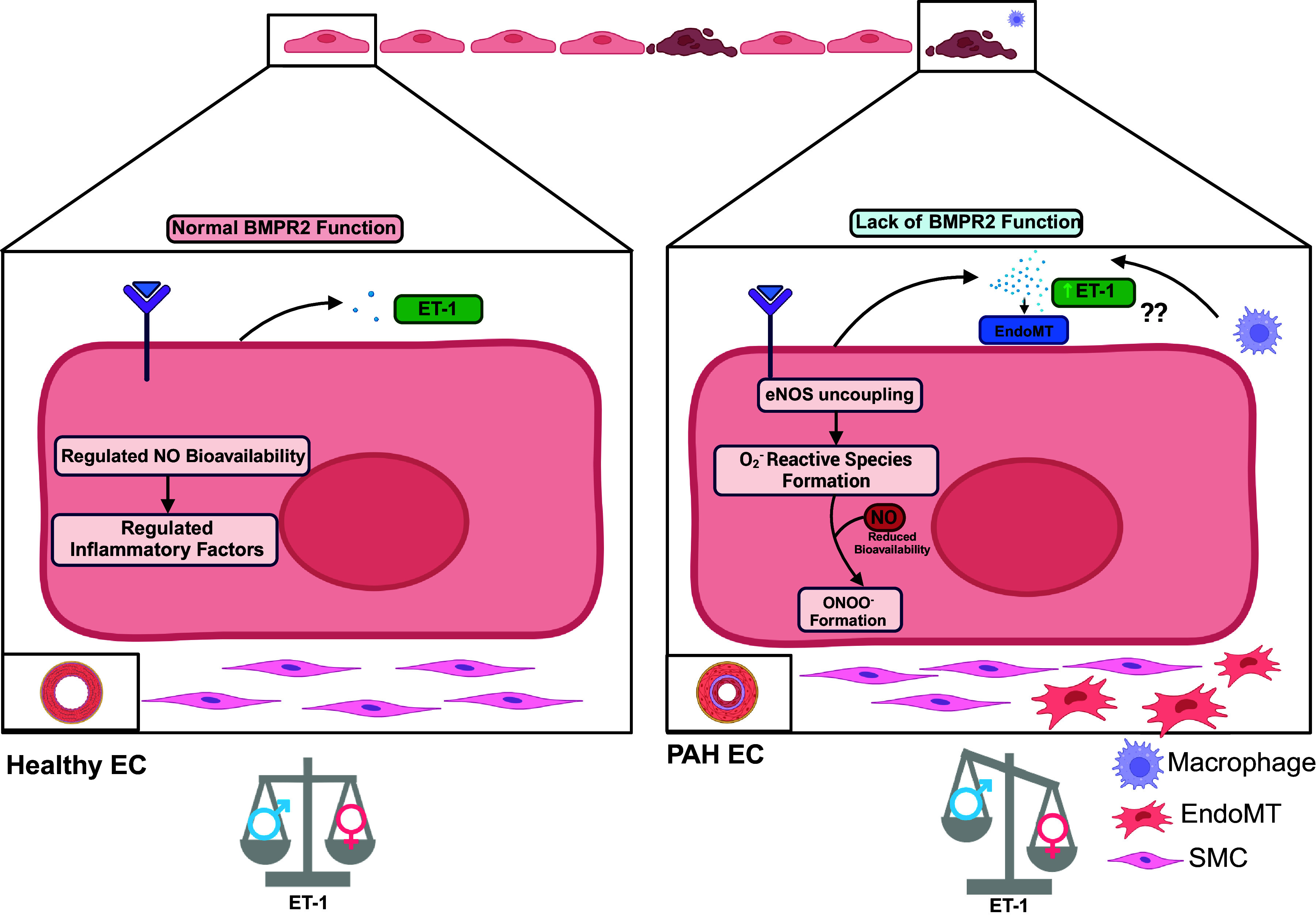
Schematic figure showing healthy lung endothelial cells (EC) vs. pulmonary arterial hypertension (PAH)-associated EC. On the left, we have a healthy EC with normal BMPR2 function. These cells will produce normal levels of ET-1 as well as regulated synthesis of NO, which will yield regulated inflammatory factors that control the inner lining of blood vessels. We also have a normal functioning SMC and similar levels of ET-1 between males and females. On the right, a PAH EC can be seen. In this cell, the BMPR2 function is reduced. Also, this cell will produce more ET-1, determining EndoMT. It is also possible that macrophages contribute to the elevated levels of ET-1. Within the cell, it can be observed that there is eNOS uncoupling, which results in $O_{2}^{-}$ reactive species that, when bound with available NO, produces ONOO^−^. Elevated reactive nitrogen and oxygen species contribute to significant inflammation within and around the pulmonary blood vessels. In addition, EndoMT induced by ET-1 may result in the development of a mesenchymal phenotype, which will play a role in the inflammation and vasoconstriction of the blood vessels. It is also observed that with the BMPR2 mutation, females have higher levels of ET-1. BMPR2, bone morphogenetic protein receptor type II; eNOS, endothelial Nitric Oxide Synthase; NO, nitric oxide; ONOO^−^, peroxynitrite; ET-1, endothelin-1; EndoMT, endothelial-to-mesenchymal transition. Figure created with BioRender.com.

In 2015, Satwiko et al. (53) observed that ET-1 transgenic mice with elevated levels of ET-1 in their lungs showed increased RVSP and pulmonary arterial (PA) thickness when exposed to chronic hypoxia. Recent studies observed a sex-linked elevation of lung ET-1 expression in a spontaneous murine model of PH (aged *BMPR2^+/R899X^* mutants) (5); however, the specific molecular mechanism underlying this effect is not completely clear yet (Fig. 2). Reduced BMPR2 expression has also been implicated in alterations in the synthesis of vasoactive molecules including NO (54) and eNOS expression in female animals exposed to hypoxia (5). Interestingly, decreased NO synthesis was also observed as a result of shear stress-induced Piezo2 depletion, a gene linked to blood vessel formation and structure, resulting in pulmonary vascular remodeling (55). A similar depletion of Piezo2 was identified in BMPR2 loss-of-function mutant animal models, indicating that loss of BMPR2-mediated signaling can alter NO synthesis through defective Piezo2 expression (55), introducing another putative target for future restoration of the lung vascular endothelial-derived NO level.

## THE INFECTIOUS ORIGIN OF LUNG EC INFLAMMATION AND RESPONSE TO PATHOGENS

### Parasites: *Schistosoma Spp*

The genus *Schistosoma* contains intravascular parasites or flatworms that are called schistosomes (56). Among the species, *Schistosoma mansoni* is the most common species capable of inducing severe PAH. After migrating through the cardiovascular system, adult *S. mansoni* enters the portal venous system and mesenteric circulation, releasing thousands of eggs capable of causing chronic injury to many organs in a disease called Schistosomiasis (2). One final complication of chronic *S. mansoni* infection is the development of Sch-PAH. Globally, Sch-PAH is the most common form of PAH, and yet it has no cure (14). The lung pathology influenced by infection is hypothesized to be caused by the embolization of *S. mansoni* eggs in the lungs, eventually leading to lung inflammation and vascular remodeling (3). Specifically, *S. mansoni* egg infection contributes to pulmonary vascular plexiform lesions, obstruction, and Type 2-associated inflammation (14, 57).

Similarly, another species, *S. japonicum*, is also implicated in inducing a milder form of PH than infection with *S. mansoni* (57). Researchers suggest this difference may be influenced by the tendency of S*. japonicum* eggs to cluster in tissues compared with the solitary distribution of the *S. mansoni* eggs (57); however, this mild effect could also be due at least in part to immunological and genetic coevolutive differences between these parasites and their host. Furthermore, a recent study on *S. haematobium* infection also observed a milder form of PH similar to *S. Japonicum* infection compared with *S. mansoni* infection (58). Interestingly enough, *S. haematobium* is unable to cross-sensitize for *S. mansoni* even though they are closer genetically related than *S. Japonicum,* which can cross-sensitize with *S. mansoni* (58). Overall, literature data heavily describes the impact of *S. mansoni* infection on PH, demonstrating the existence of a research gap in understanding how other species contribute to the development of the disease.

### Viruses: HIV and Severe Acute Respiratory Syndrome Coronavirus 2

Beyond parasitic infections, viruses such as HIV also cause lung EC injury that chronically can evolve into PAH (3). In the lungs, HIV-mediated EC injury seems highly dependent on virus-associated proteins such as Nef, Tat, and gp120 (3, 59) along with pro-inflammatory factors, including Endothelial-Monocyte Activating Polypeptide II (60, 61). Tat and Nef proteins have been associated with a senescence phenotype during HIV infection by increasing oxidative stress, which can lead to age-related inflammation and disrupt DNA repair (62). In terms of the PAH, both oxidative stress and endothelial senescence have been implicated in the development and progression of disease (63). This information may indicate a potential relationship between HIV-induced cellular senescence and the development of PAH. HIV infection is not the only virus associated with lung EC injury; severe acute respiratory syndrome coronavirus 2 (SARS-CoV-2), the COVID-19 infectious agent, has also been implicated in the predisposing of the development of PAH (64). However, it remains unclear how SARS-CoV-2 damages lung ECs, with discussions about whether by direct viral infection or indirectly by inflammatory mediators (65). Furthermore, PH imposes a risk for COVID-19 disease outcome, with a higher fatality rate observed for patients with COVID-19 who have PAH than the general population (66), raising awareness of the potential negative impact of new pathogenic threats for patients with PAH. Besides reported that lung endothelial damage does not occur via direct viral infection [even with their significantly elevated levels of human angiotensin-converting enzyme 2 (hACE2) in this cell type (65, 67)], it seems like EC response to viral proteins such as S-proteins, responsible for receptor recognition and cell membrane fusion (68, 69), may contribute to the observed lung vascular “endothelialitis” (70). Furthermore, SARS-CoV-2 can activate Interferon-β release, which is associated with antiviral effects and impaired endothelial response, in SARS-CoV-2 infected airway epithelium (71). This study suggests that viral-induced Interferons may modulate EC senescence, which may contribute to an antiviral effect but also promote tissue injury. Finally, these recent studies investigating cellular senescence and endothelial damage are uncovering the potentially harmful relationship between viruses such as HIV and SARS-Cov-2 and the progressive development of PAH.

### Fungi: *Pneumocystis, Paracoccidioides Lutzii*, and *Cryptococcus neoformans*

Different Fungi species have also been implicated in lung EC injury. For example, Pneumocystis, induced by infection with *Pneumocystis* colonization, can promote inflammatory responses, endothelial dysfunction, and PH (72). In one study, *Pneumocystis*-associated PH has been observed to have increased vasoconstrictor response to ET-1 and fungi colonization (72). *Pneumocystis* is frequent in patients with chronic obstructive pulmonary disease (COPD) and produces local and systemic inflammation (72). However, pneumocystis is not the only fungus associated with lung endothelial damage; paracoccidioidomycosis is also known to be associated with endothelium. Paracoccidioidomycosis is caused by an infection of *Paracoccidioides lutzii*, which can evolve into severe cardio-respiratory complications, including PAH, due to tissue necrosis and fibrosis (73). In terms of *Cryptococcus neoformans* infection, one study observed that deletion of an uncharacterized protein CSN120 blocked pulmonary infection and dissemination of the infections, suggesting it might be required for pulmonary invasion of the fungi (74). It was also observed that mutant CSN120 mice developed granuloma formation in the early stage of infection, whereas CSN120 deletion alleviated inflammation and expansion of the *Cryptococcus neoformans*-induced infection (74). Overall, the role of fungi infection on EC and vascular diseases remains a potential and promising avenue for further research endeavors.

### Bacteria: *Escherichia coli*, *Streptococcus pneumoniae*, and *Staphylococcus aureus*

In terms of bacteria, excessive production of pro-inflammatory mediators, like those observed during sepsis, is known to promote lung EC dysfunction and vascular inflammation. Not only pro-inflammatory soluble mediators but circulating extracellular vesicles (EVs) also influence intercellular communication, especially among ECs, immune cells, and pathogens. In a sepsis model, EVs were associated with the Gram-negative bacteria *Escherichia coli* (75). Specifically, *E. coli*-mediated activation of lung endothelial TLR4/MyD88/IRAK-1 and p38 signaling pathways repressed Endothelial Ribonuclease 1, known to be protective against extracellular RNA-mediated cell damage and inflammation (75). In another study, EC’s efficiency in capturing *E. coli* over time was affected by neutrophil depletion (76), demonstrating the complexities of lung intercellular communication during infection.

Gram-negative bacteria are not the only ones capable of modulating EC function; Gram-positive bacteria can also impair lung endothelial function (77). For example, *Streptococcus pneumoniae* infection is associated with lung vascular dysfunction in mice by impairing lung compliance via overexpression and function of Endothelial Protein C Receptor (EPCR) (78). In addition, one case study from 2022 also reported the development of severe PH in a neonate after infection with *S. pneumoniae* (79). Similarly, another Gram-positive bacterium*, Staphylococcus aureus,* also causes lung EC barrier dysfunction via group V phospholipase A2 (gVPLA2)—implied as a potential novel therapeutic target in methicillin-resistant *S. aureus* (MRSA) by diminishing MRSA-induced permeability in lung ECs (80). Another study also demonstrated *S. aureus’s* contribution to lung EC barrier dysfunction and inflammation, but mechanisms are still not fully understood (81). Detrimental effects of this bacteria can be triggered by the activation of mitogen-activated protein kinase (MAPK), extracellular signal-regulated kinase ½ (Erk ½), and nuclear factor kappa-light-chain-enhancer of activated B cells (NF-*κ*B) inflammatory cascade and small GTPase RhoA, which contribute to cytoskeletal remodeling and endothelial barrier integrity (81). This study also described that heat-inactivated *S. aureus* induced destabilization of the EC cytoskeleton, increasing cell permeability and inflammation (81).

### Host Innate Microbes: Role of the Host Gut and Lung Microbiome

Until recently, it was utterly unknown whether the composition of the host microbiome would contribute to PAH. However, the field has been rapidly progressing toward a better understanding of the intersection of host microbes and PAH. In terms of chronic PAH-linked infection, *S. mansoni eggs* were observed to significantly alter the gut and the lung microbiome, leading to pulmonary vascular remodeling, EC injury, and expansion of an abnormal EC phenotype (14). Acutely, there has been evidence between the gut microbiota and the development of ALI-induced vascular injury (82). Indeed, the gut microbiome can affect systemic inflammatory response (83), but no conclusive molecular mechanism has yet clearly demonstrated how the gut microbiome directly affects PAH onset and progression (83). What is known based on recent reports is that patients with PAH have a proinflammatory gut microbiome marked by reduced alpha diversity. Moreover, most gut microbiota identified expressed similar differences between groups, but the gut virome indicated interesting differences (84). Finally, functional changes in the gut microbiome were investigated by LEfSe (Linear discriminant analysis Effect Size) analysis. Amino acids synthesis was enriched by pathway analysis, with increased arginine, proline, ornithine biosynthesis, and interconversion as one of the main functional changes in the altered PAH gut microbiome. At the genus level, *Blautia* and *Bifidobacteria* mostly contributed to arginine/ornithine biosynthesis in the PAH gut microbiome, whereas *Collinsella* contributed to increased proline biosynthesis (84). These data suggest the potential roles of enzymes and amino acids produced by the microbiota in PAH and cardiovascular disease risk, opening up novel therapeutic avenues for treating this life-threatening disease (Table 1).

**Table 1.** Association between pathogens, endothelial cells, and pulmonary arterial hypertension

Types of Pathogens	Endothelial Cell Impact	Pulmonary Arterial Hypertension Impact
Parasites
*S. mansoni*	EC leukocyte adhesion and solitary-egg distribution (57)	Severe form of PH (57)
*S. haematobium*	Unclear EC impact (58)	Milder form of PH similar to *S. japonicum* (58)
*S. japonicum*	EC egg cluster distribution and smaller granulomas than the *S. mansoni* (57)	Milder form of PH similar to *S. haematobium* (57)
Viruses
HIV	EC activation and apoptosis (3)	PAH onset and progression (61)
COVID-19	EC oxidative stress and fibrosis (66)	Predisposition to PAH and higher fatality rate for patients with PAH (64, 66)
Fungi
*C. neoformans*	Dissemination of infection (74)	Unclear relationship with PAH
*Pneumocystis*	Vasoconstrictor response (72)	Associated with PH (72)
*P. lutzii*	Necrosis and fibrosis (73)	May lead to PAH (73)
Bacteria
*E. coli*	Infection mediated by EC (76)	Unclear relationship with PAH
*S. aureus*	EC barrier disruption (80)	Unclear relationship with PAH
*S. pneumonae*	Impaired lung compliance (78)	May cause respiratory illnesses and there is a case report of the infection leading to PAH (78, 79)
Microbes
Gut microbiome	Can improve EC injury (82)	There is a link between the gut microbiome and Sch-PAH (14)

EC, endothelial cell; PH, pulmonary hypertension; PAH, pulmonary arterial hypertension; Sch-PAH, schistosomiasis-associated pulmonary hypertension.

## OTHER CELL TYPES AND INDIRECT MEDIATORS

Other vascular cells like SMCs, fibroblasts, and pericytes also contribute to EC injury and PAH progression. Primarily, the crosstalk between ECs and SMCs stands out as fundamental for maintaining vascular homeostasis, with the uncontrolled proliferation of SMCs resulting in thickening of pulmonary arteries and disease progression. Many studies have been conducted to uncover the underlying mechanisms of this proliferative shift. One key factor recently identified is Forkhead box M1 (FoxM1), which mediates SMC proliferation and vascular remodeling through EC dysfunction (85). FoxM1 is a cell cycle regulator required for G1/S and G2/M transitions as well as M-phase progression, and its inhibition exacerbated PH in rodent models of the disease (85). Similarly, activation of PA adventitial fibroblasts (PAAfs) in response to injury and matrix stiffness also regulates vascular remodeling in PAH (86), with their derived factors such as the Fibroblast Growth Factor-23 (FGF-23) noted to influence patient outcomes to treatment responses (87). Potential therapeutic targets like sodium dichloroacetate, a pyruvate dehydrogenase kinase inhibitor, have also been shown to inhibit cell proliferation and apoptosis resistance of RV fibroblasts. RV fibrosis of patients with PAH reduces RV compliance and is associated with poor outcomes with RV pressure overload activating RV fibroblasts (88). Finally, poor EC-pericyte crosstalk is also detrimental to PAH. For example, it can reduce pericyte coverage of the pulmonary microvessels via defective Wnt/PCP activity (89). In addition, hyperproliferation and migration of vascular pericytes have been demonstrated to excessively cover the pulmonary arterioles in PAH (90); a mechanism linked to C-X-C motif chemokine receptor-7 (CXCR) and TGF-BRII signaling (90). Pericytes’ association with lung EC also contributes to their survival by transmitting HIV to susceptible cells; however, it remains unclear how HIV infection influences pericyte function, contributing to the development of chronic lung vascular disease (91).

Nonvascular cells such as macrophages are one of the primary effectors of inflammation in pulmonary vascular lesions and can lead to the progression of the disease (92). They also have been observed as the most frequent leukocytes in the RV (92) with chemokines and chemokines receptors, such as CCL2-CCR2 and CCL5-CCR5, involved in their recruitment during PAH. Specifically, the M2 macrophage phenotype significantly stimulated PASMC proliferation (93), supporting findings that macrophage depletion and/or inactivation can be targeted to prevent PH (94). Furthermore, neutrophils have also been observed to play a role in PAH, specifically with the release of NE, which is implicated in PAH arteriopathy. It was observed that NE is increased in this disease, which suggests that excessive NE levels contribute to PAH as well as an indicator of clinical status. Interestingly, NE levels have also been inversely correlated with BMPR2 expression. However, this study was unable to see whether circulating NE reflects activity in the pulmonary vasculature and the effects of current PAH therapeutics on NE release (19, 95). Overall, macrophages have been identified as powerful inflammatory effectors in pulmonary lesions, and neutrophils have emerged as potential predictors of many cardiovascular diseases.

Patients with PAH have been shown to display increasing numbers of circulating EVs (96, 97), correlated with pulmonary vascular resistance, functional impairment, and mortality (97). More than their circulating levels, their cargo, including miRNAs, are crucial for cell communication and PAH (97). EVs can be derived from many cell types, including mesenchymal stem cells, which have been described as very effective at preventing and reversing PH in the Hypoxia/Sugen rat model by reversing increased RVSP and vascular remodeling (98). EVs can also derive from shear stress-induced platelet activation, contributing to the secretion of pro-inflammatory and prothrombotic mediators, and further promoting vascular remodeling and PAH (99). Interestingly, clinical studies showed that patients treated with prostacyclin analogs had decreased platelets-derived EV shedding compared with controls (99). As research on EVs remains unfolding, its cargo and mechanism of shedding are not fully clear. In terms of the mechanism of shedding, phosphorylation of the lung EC-associated protein Caveolin-1 is a promising mechanism to be further explored. Largely known as a contributor to pulmonary vascular injury, inflammation, and progressive remodeling in PAH, lung endothelial Cav-1 depletion in PAH partially occurs via the shedding of EVs, contributing to TGF-β secretion from bone marrow-derived macrophages in PAH (96). On the contrary, adipose-derived mesenchymal stem cells and -derived exosomes appear to exert beneficial effects in PAH by reducing inflammation and cell death (100). Finally, specific miRNA EV cargo such as miR-191 has been observed to repress BMPR2 and mediate human PAEC proliferation, although the mechanism remains under investigation (100).

## CONCLUSIONS

PAH-associated EC phenotype arises from a variety of mechanisms, including as a consequence of EndoMT or apoptosis-resistance. The expansion of these phenotypes has been linked with the depletion of key molecules expressed in the pulmonary endothelium, including BMPR2. Crucial for lung EC survival, BMPR2 depletion is largely implicated in the development of multiple subgroups of PAH, including pathogen-associated. Similarly, reduced NO, elevated levels of ET-1, and indirect messengers like EVs play important roles in disease onset, progression, and severity. Whereas lung ECs are often at the center of the initiating mechanisms linked to persistent vascular injury leading to remodeling in PAH, other cell types also significantly influence the disease’s progression. Moreover, other organisms such as parasites, bacteria, fungi, and viruses can disrupt lung EC function, contributing to the development of acute and chronic vascular diseases such as PAH. Whereas the link between some infectious agents, like *S. mansoni*, has been more clearly established in PAH, whether a precise molecular mechanism is a common denominator in driving the disease remains not entirely clear. Similarly, little is known about the direct impact of gut and lung microbiome dysbiosis on lung endothelial function, leaving many research avenues widely open for progressively building a more solid research basis for future clinical trials leveraging microbes or targeting pathogens associated with PAH. The studies discussed in this review provide a foundation for further investigation into the relationship between lung EC survival and the development of PAH, highlighting numerous potential targets and pathways in ongoing and for future research. Specifically, data summarized here highlighted signaling pathways and processes related to a lung abnormal EC survival observed in PAH, including mediated by BMPR2 reduction and mutation, eNOS dysfunction, perivascular inflammation, and the role of infectious agents. Understanding the complex connections among these pathways provides a broad range of potential novel lung vascular EC-targeted therapies. For example, exploring the influence of the lung microbiome composition in health models and patients and uncovering how infection affects its dynamics along with the contribution of specific microbial functional groups or species reduced or increased in the lungs of patients with PAH, enables novel biotherapeutic approaches to revert or ameliorate this life-threatening disease. In addition, repurposing and developing novel specific EC apoptosis inhibitors could rescue the equilibrium among vascular cell phenotypes and prevent expansion of uncontrolled proliferative ECs in PAH. As mentioned before, PAH is a progressive disease with sex bias, and thus, further studies evaluating the impact of sex and hormonal therapies on lung EC survival would also be crucial for the development of novel sex-target therapeutic approaches.

## GRANTS

This work was supported in part by funding from the National Institute of Health/National Heart, Lung, and Blood Institute (HL159037) and the UIC Health Equity Pilot Program (HEPP) Award on Women-Prevalent Disease Research (to S.D.O.); scholarship support from a UIC Liberal Arts and Science Undergraduate Research Initiative (LASURI) and the Honors College Research Grant (to E.S.V.); and scholarship support from UIC Latin@s Gaining Access to Networks for Advancement in Science (L@S GANAS) and UIC Chancellor’s Undergraduate Research Award (CURA) (to O.L.).

## DISCLOSURES

No conflicts of interest, financial or otherwise, are declared by the authors.

## AUTHOR CONTRIBUTIONS

S.D.O. conceived and designed research; Y.M., E.S.V., and O.L. prepared figures; Y.M., E.S.V., O.L., and S.D.O. drafted manuscript; Y.M. and S.D.O. edited and revised manuscript; S.D.O. approved final version of manuscript.
